# The Effect of Animal Movement on Line Transect Estimates of Abundance

**DOI:** 10.1371/journal.pone.0121333

**Published:** 2015-03-23

**Authors:** Richard Glennie, Stephen T. Buckland, Len Thomas

**Affiliations:** Centre for Research into Ecological and Environmental Modelling, University of St Andrews, St Andrews, UK; Université de Sherbrooke, CANADA

## Abstract

Line transect sampling is a distance sampling method for estimating the abundance of wild animal populations. One key assumption of this method is that all animals are detected at their initial location. Animal movement independent of the transect and observer can thus cause substantial bias. We present an analytic expression for this bias when detection within the transect is certain (strip transect sampling) and use simulation to quantify bias when detection falls off with distance from the line (line transect sampling). We also explore the non-linear relationship between bias, detection, and animal movement by varying detectability and movement type. We consider animals that move in randomly orientated straight lines, which provides an upper bound on bias, and animals that are constrained to a home range of random radius. We find that bias is reduced when animal movement is constrained, and bias is considerably smaller in line transect sampling than strip transect sampling provided that mean animal speed is less than observer speed. By contrast, when mean animal speed exceeds observer speed the bias in line transect sampling becomes comparable with, and may exceed, that of strip transect sampling. Bias from independent animal movement is reduced by the observer searching further perpendicular to the transect, searching a shorter distance ahead and by ignoring animals that may overtake the observer from behind. However, when animals move in response to the observer, the standard practice of searching further ahead should continue as the bias from responsive movement is often greater than that from independent movement.

## Introduction

Line transect sampling is a distance sampling method [1, 2], widely used for estimating the abundance of wild animal populations. Lines are placed randomly across a study region, or more usually a systematic grid of lines is randomly positioned, and the observer traverses each line, recording the perpendicular distance from the line to each detected animal. Typically, the observer fails to detect all animals in the surveyed strip, and these distances are used to model the probability of detection, and hence to estimate abundance.

The method relies on three key assumptions, and if these are not met then estimates of abundance can have substantial bias [1] (pp. 29–37). These are:

1. animals on the line are detected with certainty;
2. measurements are exact;
3. animals are detected at their initial location.

When the first assumption is not met, double-observer (or multi-observer) methods, which combine distance sampling with mark-recapture, may be used [3, 4]. Similarly, if the second assumption is violated, measurement error models [5, 6] can be adopted. We concentrate on violations of the third assumption.

Conceptually, line transect sampling is a snapshot method [1] (pp. 31), that is, we consider that the animals are at a fixed location while the survey takes place. Thus, we have probabilistic encounters between a moving observer and immobile animals [7]. There are models [8, 9] for encounters between mobile animal populations and observers, but these are limited in use: they rely upon quantities that are difficult to determine (mean animal speed, encounter radius) and they assume an ideal free gas movement model which can be unrealistic [10]. Making the third assumption avoids such problems. This assumption can be violated in two ways: animals can move in response to the observer, or move independently of the observer. Responsive movement is a common problem in distance sampling surveys and the bias it causes can be reduced by modelling the movement [11, 12] or using double-observer methods [13]. In a few specific cases, independent movement has been modelled (for fish: [14]; for seabirds: [15]) to reduce bias; however, these methods are *ad hoc* and specific to their application. We do not explore responsive movement here; instead, we consider how animal movement independent of the observer affects bias. For whale surveys, it was concluded that such movement may seriously bias the estimate of mean density if the speed of the whales approaches that of the observer, if the encounter region is long rather than wide, or if the probability of sighting a whale is not strongly dependent on the time it spends within that region [16]. Subsequently, simulation revealed that bias was negligible if mean animal speed was one quarter of that of the observer, but not if animal speed was one half that of the observer [17]. This conclusion has now been adopted within distance sampling [1] (pp. 131, 173) and is used to determine if independent animal movement is a problem in particular studies [18]. This is despite these results being pertinent only to the single simulation scenario considered.

In this paper, we quantify the bias caused by independent animal movement in some example cases and discuss the non-linear relationship between movement, detection and bias. We first consider strip transect sampling which is a special case of line transect sampling in which all animals within a strip are assumed to be detected [1] (pp. 2–3). We derive an analytic expression for the bias caused when animals move in straight lines, each with the same constant speed but an individual random direction, within a rectangular study area. We then generalize to surveys in which detectability falls off with distance from the line, and for which animal movement is more complex, using simulation to quantify bias. Finally, we discuss what these results reveal about bias caused by animal movement independent of the observer.

## Strip Transect Sampling

Consider estimating the abundance of an animal population using strip transect sampling where the study area is rectangular of length *a* and breadth *L* with corners, in (*x*, *y*)-coordinates, at (−*a*/2, 0), (*a*/2, 0), (−*a*/2, *L*), (*a*/2, *L*). We assume the number of animals within the study area is fixed, and so adopt a wrap-around model: for each animal that exits across one boundary of the study area, a new animal enters immediately at the same distance along the opposite boundary. Intuitively, we think of the study area as part of a wider region. We assume the (strip) transect is placed randomly within this region and so may take the transect to be at *x* = 0. Let the transect have half-width *w*, where 2*w* < *a*, extending out to either side of *x* = 0 and length *L*, so it transverses the entire study region in the *y*-direction.

**Fig 1:**
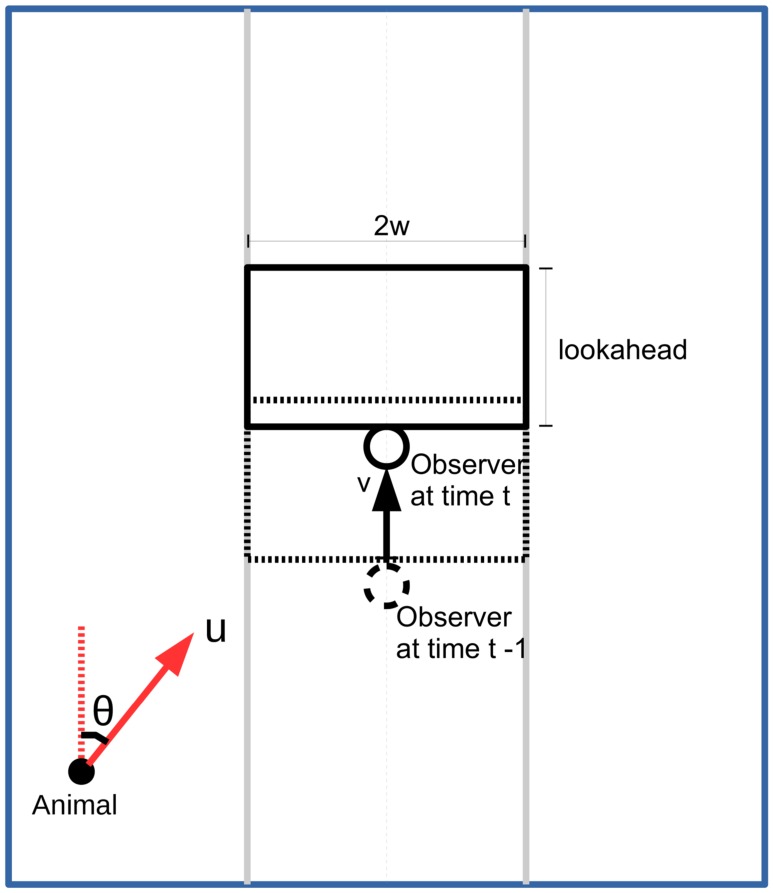
Strip transect with encounter region. Transect line (dashed grey line) with the strip extending a distance *w* out to either side (solid grey lines). The observer and encounter region at time *t* − 1 (dashed black) and *t* (solid black) are shown, together with an animal moving with speed *u* in direction *θ*

We assume animal *i* travels in a straight line at speed *u* and in a direction *θ*_*i*_ where *θ*_*i*_ is a random deviate from a uniform distribution *U*(0, 2*π*] (Fig. 1). Thus, the equations of motion of the *i*th animal are given by:

$$\begin{array}{ccc} y_{i,t} & = & y_{i,0}+u\cos\left(\theta_{i}\right)t\\ x_{i,t} & = & x_{i,0}+u\sin\left(\theta_{i}\right)t \end{array}$$

where *t* is time and (*x*_*i*, 0_, *y*_*i*, 0_) is the initial (*t* = 0) location of animal *i*.

The observer travels along the centerline, *x* = 0, at speed *v* > 0, starting at *y* = 0. Thus, the position of the observer at time *t* is given by (0, *z*_*t*_) where *z*_*t*_ = *vt*. We define the *encounter region* to be the area around the observer within which every animal is recorded. No animals are recorded outside the encounter region. Here, we assume the encounter region is rectangular, extending a distance *w* either side of the centerline and a distance *l*, which we call the lookahead distance, in front of the observer (Fig. 1). The encounter region is wrapped using the wrap-around model to fix its size. (There are practical methods of implementing a wrap-around design with strip transects, using buffer zones [19].)

When estimating abundance using strip transect sampling, it is assumed that the animals are immobile—this is termed a *snapshot* method as the survey is assumed to take place instantaneously [1] (pp. 31). When true, the expected proportion of animals within the strip is $P_{1}=\frac{2w}{a}$. However, if animals are mobile then they can move into the encounter region and so be recorded; hence, we expect to record more animals, which causes positive bias in abundance estimates.

Animals not initially inside the encounter region can enter in three ways: from in front, from the side and from behind. We shall consider each in turn. First, bias is not caused by animals travelling toward the observer and entering the encounter region from in front since on average in any interval of time an equal number of animals, that if immobile would be recorded, will move away from the observer and not be recorded. Second, bias is caused by animals entering the encounter region from the side. Any point along the strip is within the encounter region of length *l* ahead of the observer for $\frac{l}{v}$ units of time. In that time, all animals with direction *θ* and speed *u* travel $\frac{lu\sin(\theta )}{v}$ units of distance perpendicular to the line. Hence the proportion of all such animals not initially inside the encounter region entering from the side and so being recorded is given by $\frac{lu|\sin(\theta )|}{av}$. Given that *θ* is uniform on (0, 2*π*], the expectation of this proportion is

$$\begin{array}{c} P_{2}=\frac{luE[|\sin(\theta )|]}{av}=\frac{2lu}{\pi av} \end{array}$$

as $E[|\sin(\theta )|]=\frac{2}{\pi }$.

Lastly, animals can only enter the encounter region from behind by overtaking the observer; hence, no bias is caused when animals travel slower than the observer. Under our movement model, only animals that are initially behind the transect, *y* < 0, can overtake the observer and enter the encounter region. By the equations of motion given above, an animal with direction *θ* and initial location (*x*_0_, *y*_0_) overtakes the observer at time

$$\begin{array}{c} T=\frac{y_{0}}{v-u\cos(\theta )} \end{array}$$

Thus, to have $0<T<\frac{L}{v}$ when *y*_0_ < 0 and have the animal recorded, we require that the following inequalities are satisfied:

$$\begin{array}{ccc} \frac{v}{u} & < & \cos(\theta )\\ -\frac{b}{v}\left(u\cos\left(\theta \right)-v\right) & \leq & y_{0}\leq 0\\ -w & \leq & x_{T+k}\leq w \end{array}$$

for some *k* ∈ [0, *T*_*δ*_] where $T_{\delta }=\frac{l}{u\cos(\theta )-v}$ is the time the animal is within the encounter region. From these inequalities, we see that within the subset of those animals with direction *θ* such that $\cos(\theta )>\frac{v}{u}$, the proportion that are recorded is given by

$$\begin{array}{c} \frac{\left(2w+u|\sin\left(\theta \right)\right|T_{\delta })\left(\frac{L}{v}\left(u\cos\left(\theta \right)-v\right)\right)}{aL} \end{array}$$

The expected value of this proportion with respect to *θ* is

$$\begin{array}{c} \frac{2w\sqrt{u^{2}-v^{2}}}{\cos^{-1}\left(\frac{v}{u}\right)av}-\frac{2w}{a}+\frac{ul(1-\frac{v}{u})}{av\cos^{-1}\left(\frac{v}{u}\right)} \end{array}$$

since $E\left(|\sin(\theta )|\;|\cos(\theta )>\frac{v}{u}\right)=\frac{1-\frac{v}{u}}{\cos^{-1}(\frac{v}{u})}$ and $E\left(\cos(\theta )\;|\cos(\theta )>\frac{v}{u}\right)=\frac{\sqrt{u^{2}-v^{2}}}{u\cos^{-1}\left(\frac{v}{u}\right)}$. Finally, since the proportion of animals with required direction *θ* is given by $P\left(\cos(\theta )>\frac{v}{u}\right)=\frac{1}{\pi }\cos^{-1}\left(\frac{v}{u}\right)$, the total proportion of animals causing this bias is

$$\begin{array}{c} P_{3}=\frac{2w\sqrt{u^{2}-v^{2}}}{a\pi v}-\frac{2w}{\pi a}\cos^{-1}\left(\frac{v}{u}\right)+\frac{l(u-v)}{\pi av} \end{array}$$

We are now able to give an expression for the total bias in the abundance estimate. If *P* is the total expected proportion of animals detected in the strip transect, then when *u* ≤ *v*, *P* = *P*_1_ + *P*_2_ and when *u* > *v*, *P* = *P*_1_ + *P*_2_ + *P*_3_. Thus, strip transect sampling produces an abundance estimate with percentage bias given by

$$\begin{array}{c} 100\times \left\{\begin{array}{cc} \frac{ul}{\pi wv} & \;u\leq v\\ \frac{ul}{\pi wv}+\frac{\sqrt{u^{2}-v^{2}}}{\pi v}-\frac{1}{\pi }\cos^{-1}\left(\frac{v}{u}\right)+\frac{l\left(u-v\right)}{2w\pi v} & \;u>v \end{array}\right. \end{array}$$

In particular, if movement is absent (*u* = 0) or animal speed is less than observer speed (*u* ≤ *v*) and the observer only searches abeam, not ahead (*l* = 0), then there is no bias. Nevertheless, the bias caused can be substantial. For example, if we assume that the observer searches as far ahead as to the side, *l* = *w*, and the animals are slower than the observer, *u* < *v*, then bias is the ratio of animal speed to observer speed, divided by *π*. Thus, if animal speed is one third observer speed, bias is 1/(3*π*) = 10.6%.

## Line Transect Sampling

In line transect sampling, some animals in the covered strip may be undetected. Movement independent of the observer causes bias in the estimated probability of detection, and this bias combines with the bias arising from movement of animals into the encounter region, so that the formulae of the previous section no longer apply.

The detection process can be modelled as either a continuous [20, 21] or a discrete [20, 22] hazard-rate process. The discrete model is more appropriate for surveys of whales, when detection cue is primarily a whale blow, or for songbirds, if the cue is a songburst. Hiby adopted a discrete hazard-rate model [17]. To avoid introducing another parameter (cue rate), we adopt a continuous-time model.

By combining animal movement with a hazard-rate detection process, the problem of deriving an analytic expression for bias appears intractable as it is a non-linear function of the mean probability of detection. Therefore, a simulation study was performed in order to quantify bias and investigate the relationship between bias, movement and detection. We first describe the simulation setup (Section 1) and then present and discuss the results as they relate to bias (Section 2), the detection function (Section 2.1), lookahead distance (Section 2.2) and truncation distance (Section 2.3).

### 1 Simulation Setup

We consider two animal movement types at a range of speeds relative to the observer. We also vary the maximal distance the observer searches ahead (the lookahead distance) and the shape of the detection function: both control the size of the encounter region around the observer.

The study area and transect are defined similarly to the strip transect case given above. The study area was one square kilometre and a wrap-around model was used. The transect was one kilometre long and bisected the study area. There were 100 animals distributed, for each simulation, randomly across the study area.

Two types of animal movement were considered. First, the linear movement model, the same as in the strip transect case, was used, where all animals moved in straight lines at the same speed but each in a randomly assigned direction. It is particularly useful to quantify the bias arising from this model: for any other random animal movement, each animal will travel through a smaller range of perpendicular distances from the line compared with linear movement, and so bias will be less as bias depends critically upon how far an animal travels from its initial location. Thus, the bias arising from linear movement is an upper bound for the bias caused by any other type of random animal movement. The second model considered is a home range model: each animal is contained within a circular home range with a randomly assigned radius from a Gamma distribution with mean 20 and variance 11. Inside its home range, the animal performed a correlated random walk, where the direction of travel was perturbed at each time step of the simulation by adding a random deviate from a wrapped normal with zero mean and 0.1 variance. When an animal reached the boundary of its home range, it was reflected back by changing its direction of travel by *π* radians. By restricting the range of perpendicular distances from the line the animal may travel through, we expect the home range model to produce smaller bias than the linear movement model; clearly, however, this will depend critically on the radius of the home ranges. If home range radius greatly exceeds the strip half-width *w*, then the constraints to movement would have little effect on the bias. If, however, the radius is smaller than *w*, as might occur with many songbird territories, then the constraints on movement should result in appreciably lower bias. For each movement model we consider animal speeds from 0 m s^−1^ to 20 m s^−1^where all animals have the same speed. If each animal’s speed were to vary around a population mean, we expect bias would be similar (as indicated by some preliminary simulation) since bias depends strongly on the mean speed of the population rather than on an individual’s instantaneous speed.

The observer travels along the line at 10 m s^−1^ and the probability of detecting an animal is modelled as a continuous hazard-rate process. However, as simulation studies are, by nature, discrete we integrate this hazard over the time step used in the simulation to give the probability of being detected within that time step. Thus, the probability of being detected in time step *t* with the continuous radial hazard *k*(*r*) = *cr* ^−*d*^ where *c* > 0, *d* > 2 is

$$\begin{array}{c} g_{t}\left(r_{0},r_{1}\right)=1-\exp\left(-{\left(\frac{x_{t}}{\sigma^{2}}\right)}^{-b}\left(I_{\frac{x_{t}^{2}}{r_{1}^{2}}}-I_{\frac{x_{t}^{2}}{r_{0}^{2}}}\right)\right) \end{array}$$

where *x*_*t*_ is the perpendicular distance of the animal from the line at time *t*, *r*_0_ is the radial distance of the animal from the observer at the beginning of the time step, *r*_1_ is the radial distance at the end of the time step, *b* = *d* − 1, $\sigma^{b}=\frac{c\Gamma (0.5b)\Gamma (0.5)}{2\Gamma (0.5d)}$ and *I*_*r*_ is the cumulative distribution function of a Beta random variable evaluated at *r* with parameters (*b*, 0.5). Here, we use a time step of 0.05s duration. We will also consider different sizes of the encounter region by changing the shape parameter (*b* = 2, 3, 5) and the lookahead distance (*l* = 0, 5, 10, 15, 20, 25, 30). Finally, we will investigate the effect that truncating the data at the analysis stage has on bias by truncating at distances where the true detection function appoximately equals 0.05, 0.1, 0.2, 0.4, 0.6 and 0.8.

For each animal speed and movement model, we simulated one thousand surveys consisting of five hundred transects each. We did not consider any model selection: we fit a hazard-rate model only. The simulated data were analysed using the Multiple-Covariate Distance Sampling (MCDS) engine that is included in the software Distance [23]. The computer code used to perform this simulation study is included as online supporting material (S1 Code).

### 2 Simulation Results

**Fig 2:**
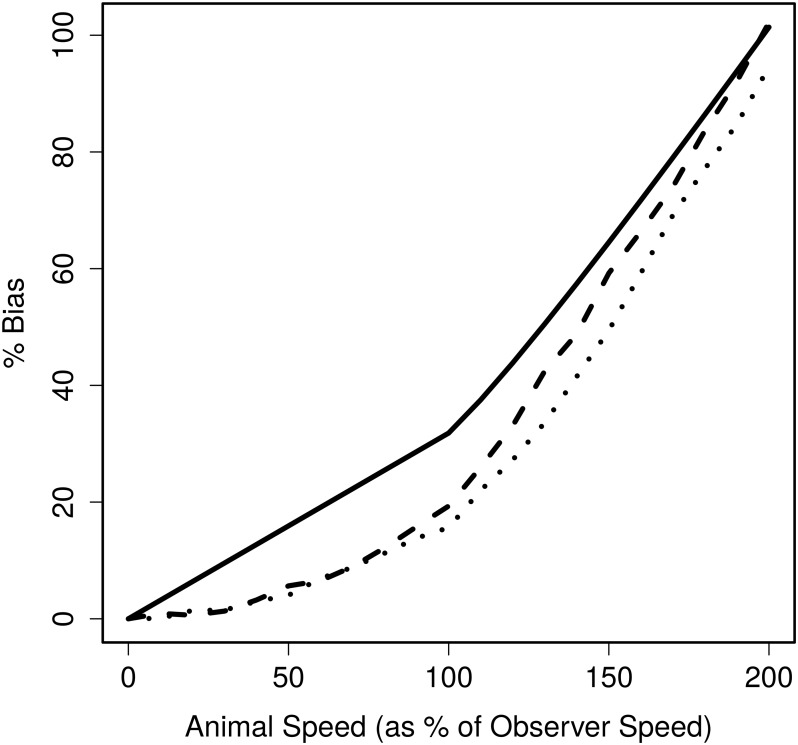
Percentage bias in the abundance estimate for strip and line transects. Bias for strip transect sampling (solid line) calculated from the analytic expression given and for line transects (hazard-rate *b* = 2) by averaging over 1000 simulations for linear movement (dashed line) and home range movement (dotted line)

Fig. 2 gives the bias estimated from the simulation study (with hazard-rate shape parameter *b* = 2) for each relative animal speed and movement model. The bias caused in strip transect sampling, calculated from the analytic expression in Section 1, with the observer searching as far ahead as to the side, is also included in the figure for comparison. The strip transect bias increases linearly when animal speed is less than observer speed, and then increases nonlinearly and rapidly thereafter. Bias in line transect sampling under both movement models behaves similarly as animal speed increases, but the bias at each speed is less than in strip transects. For strip transect sampling, bias reaches nearly 10% when animal speed is just 30% of observer speed, while for our simulation of line transect sampling under linear animal movement, bias does not reach 10% until animal speed is 80% of observer speed. However, for this scenario, at very large animal speeds (150% – 200% observer speed) the bias in line transect sampling is similar to or exceeds that in strip transect sampling.

We now consider these results. First, the difference in bias between linear movement and home range movement confirms that bias is related closely with the range of perpendicular distances from the line that an animal can travel through. At smaller speeds, the animals move through a similar range of perpendicular distances under each movement model which explains why bias is similar between the two. However, at larger speeds, animals under the linear movement model travel through a large range of perpendicular distances compared with the home range model where animals are constrained to a local territory; hence, linear movement causes more bias at these speeds. This shows that bias from animal movement is related to the distance an animal of interest may travel—the smaller that distance, the less the bias. Second, these results show that any difference in the bias in strip and line transect sampling is due to the detection process as line transect sampling is a generalisation of strip transect sampling where detection of animals is uncertain.

#### 2.1 Detection Function

**Fig 3:**
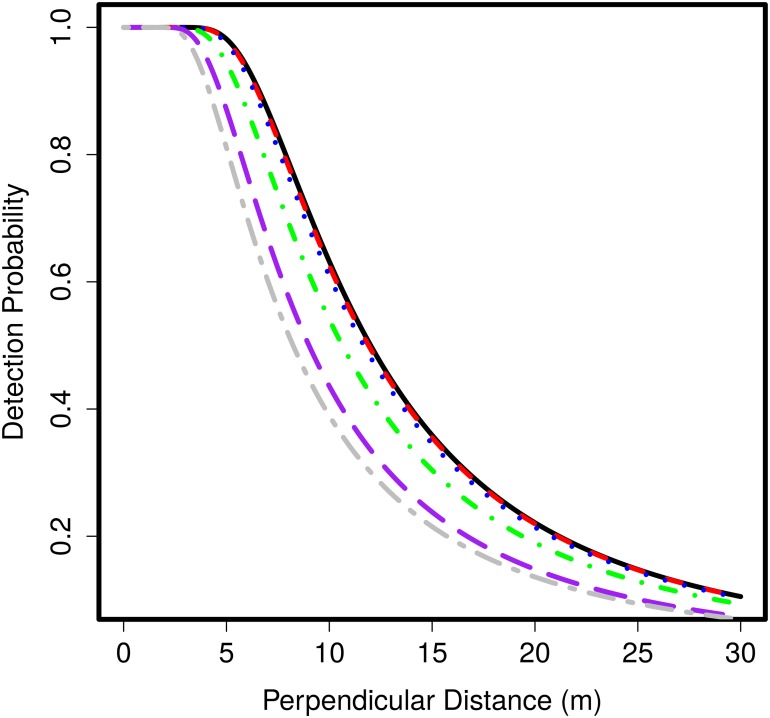
Estimated detection function for line transect sampling for increasing animal speeds. Estimated detection function averaged over 1000 simulations for line transect sampling (hazard-rate *b* = 2) with the linear animal movement model at animal speeds 0% (black solid line), 20% (red dashed line), 40% (blue dotted line), 80% (green dotdash line), 150%(purple longdash line) and 200% (grey twodash line)

**Fig 4:**
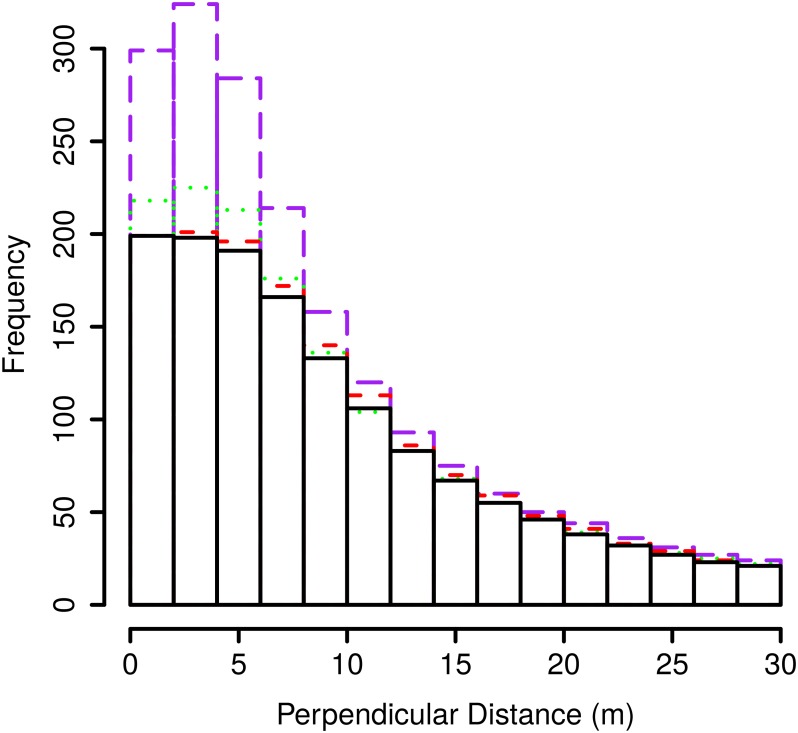
Frequency of detections at each perpendicular distance from the line. Estimated histogram of recorded distances averaged over 1000 simulations for line transect sampling (hazard-rate *b* = 2) with linear animal movement at animal speeds 0% (black solid), 20% (red dashed) 80%(green dotted) and 150% (purple dotdash)

**Fig 5:**
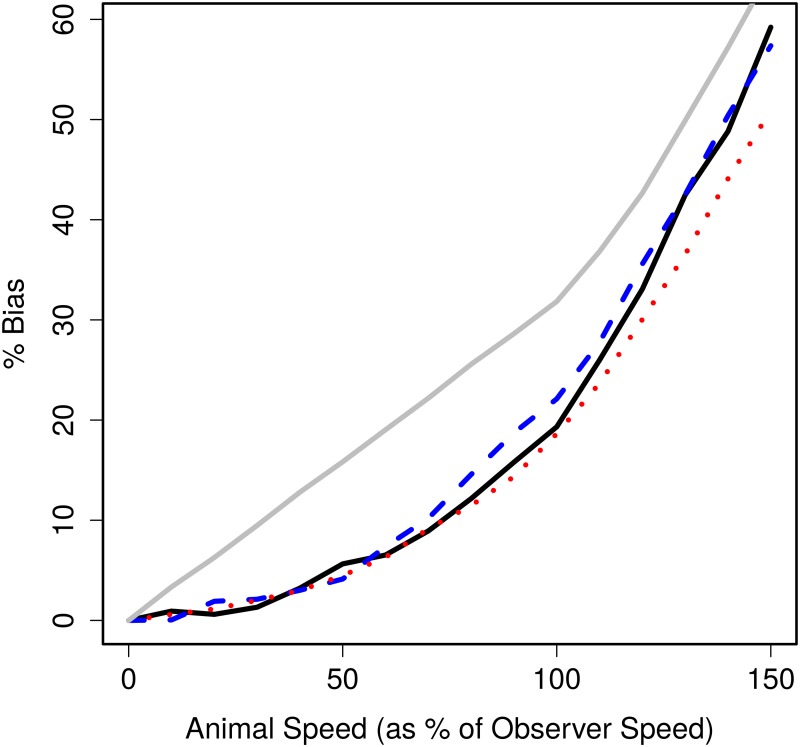
Percentage bias in abundance estimate for different shape parameter values. Percentage bias in abundance estimate for linear movement against animal speed as a percentage of observer speed for shape parameters *b* = 2 (black solid), *b* = 3 (blue dashed) and *b* = 5 (red dotted) with percentage bias for a strip transect (grey solid) estimated from 1000 simulations included for comparison.

Fig. 3 shows that as speed increases, the estimated detection probability falls off more rapidly and the shoulder (range of distances from the line for which probability of detecting an animal is one) narrows. This can also be seen in the distribution of observed distances in Fig. 4. The effect on bias of changing the shape parameter of the detection function is shown in Fig. 5. Here, we discuss how the bias in the estimated detection function occurs, how it relates to bias in abundance estimates, and how the shape parameter of the function affects bias.

Bias in the estimated detection function is a result of animal movement and the shape of the true detection function. As speed increases, the number of detections close to the line increases rapidly since more animals are able to travel very close to the line during the survey and so be detected. The number of detections at distances further from the line does not increase as rapidly since some of the animals travelling toward the line that are detected replace those that remain undetected by travelling away from the line. This does not occur very near to the line since there the probability of detection is close to one and so the animals travelling both toward and away from the line are detected. The rapid fall of detections as distance from the line increases is the reason the detection function falls more rapidly at greater speeds. This bias in the estimated detection function generates positive bias in the abundance estimate as the probability of detection is underestimated.

Nevertheless, bias in line transect estimates of abundance is less than that for strip transects when animal speed is less than 200% of observer speed. This is due to the detection process. In strip transects, animals entering the strip are detected at the boundary at which they enter since detection probability is one inside the strip and zero outside. In line transects, the animals that would be detected at the boundary in strip transects are unlikely to be detected as they approach from a distance with small detection probability. Furthermore, as detection probability falls off gradually from the line some of these animals, if detected, may replace those that moved further from the line that would, if immobile, have been recorded. So, the reduction in the number of detections by the detection process reduces bias by a greater amount than the bias in the estimated detection function augments it when animal speeds are slow. Conversely, at animal speeds close to 200% of observer speed, bias in line transect sampling becomes similar to that in strip transect sampling due to the biased estimated detection function. At these speeds, the detection function falls off very rapidly and many animals are assumed to be undetected resulting in very high bias in abundance estimates which may exceed the bias in strip transect sampling.

Bias is also related to the shape of the detection function (Fig. 5). At slow animal speeds, bias is similar for all shape parameters considered (*b* = 2, 3, 5) which is unsurprising as the bias arises from too many detections close to the observer and all three functions here have similar shapes in this region (they all approximately equal one). At animal speeds exceeding observer speed, the hazard rate with shape parameter *b* = 5 consistently produces smaller bias than the other two shape parameters (*b* = 2, 3). This may be due to the assumed detection function being isotropic: animals overtaking the observer are less likely to be detected ahead of the observer when *b* = 5 than *b* = 2, 3 since the shoulder of the detection function is narrower. However, here we not only change the shape of the detection function but also the probability of detection and so the bias may be a result of both effects.

#### 2.2 Lookahead Distance

**Fig 6:**
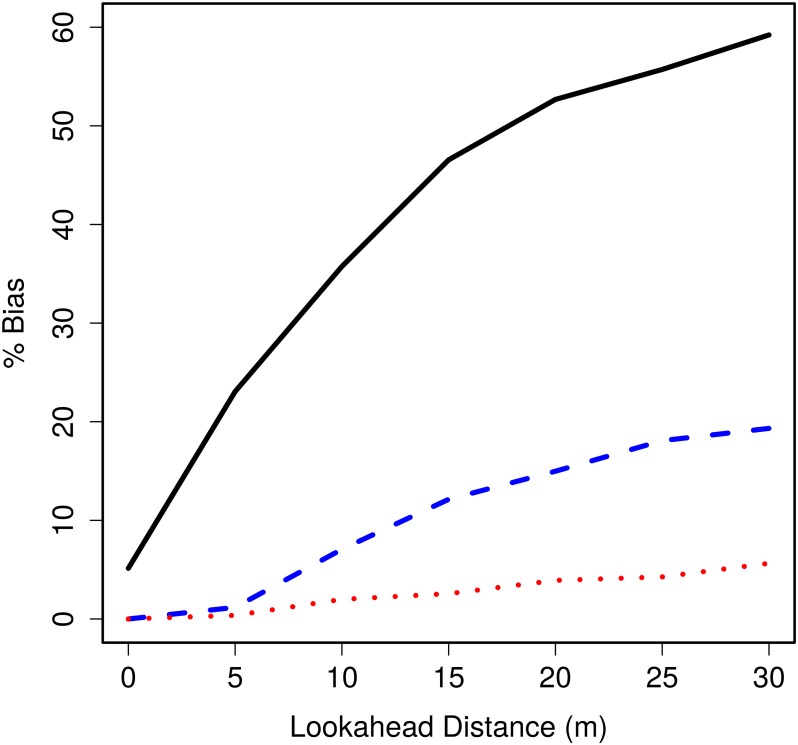
Percentage bias in abundance estimate against distance observer looks ahead. Percentage bias against distance observer looks ahead with hazard rate *b* = 2 and animal speeds 50% (red dotted), 100% (blue dashed) and 150% (black solid) of observer speed

Fig. 6 shows that bias, at each speed, increases toward a plateau as lookahead distance increases for a fixed detection function. The bias reaches a plateau since the probability of detection at a large lookahead distance is small and so further increasing lookahead distance results in a smaller increase in detections—and so bias increases by less. Also we see for small animal speeds, the bias increases slowly with lookahead distance, while for animal speeds that exceed observer speed, bias increases rapidly. As for strip transect sampling, this rapid increase is due to animals overtaking the observer from behind. For larger lookahead distances, such animals remain in the encounter region for longer and are thus more likely to be detected. Non-zero bias when the observer searches abeam (lookahead distance is zero) is another consequence of animal speed exceeding observer speed, when animals travel slower than the observer no bias occurs.

#### 2.3 Truncation Distance

When analysing line transect data, it is common to truncate the recorded distances at some truncation distance to prevent outliers unduly influencing the abundance estimate [1] (pp. 103–108). We explored the effect of truncation distance on bias with the linear animal movement model and *b* = 2 hazard-rate. We assumed that observers were actively searching out to distances beyond the truncation distance, and recorded distances of detected animals from the line when they were first detected. Thus, truncation was assumed to be applied by the analyst, not in the field.

**Fig 7:**
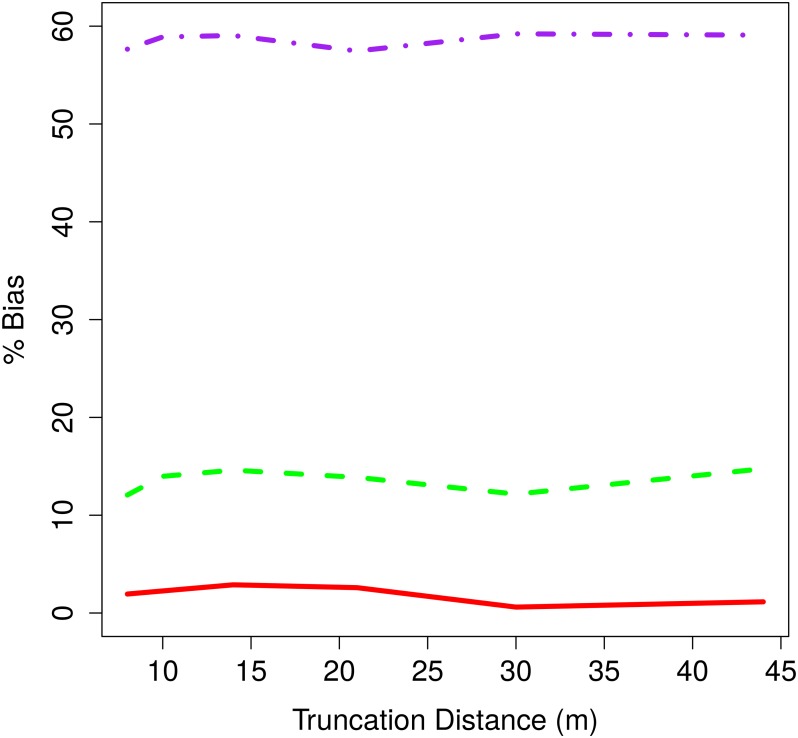
Percentage bias in abundance estimate for different truncation distances. Percentage bias in abundance estimate averaged over 1000 simulations for hazard-rate *b* = 2 against truncation distance applied by the analyst for animal speeds 20% (red solid), 80% (green dashed) and 150% (purple dotdash)

Results indicate little if any relationship between bias and truncation distance (Fig. 7). This is unsurprising as bias is caused by too many detections near to the line which truncation does not affect.

## Discussion

Animal movement independent of the observer generates bias in abundance estimates from line transect surveys. The degree of bias depends both on animal movement and dectectability. For a given mean animal speed, the analytical strip transect expression provides an upper bound for this bias across all possible detection functions and movement types. The expression shows bias to be reduced when the observer searches further to the side and a shorter distance ahead (smaller lookahead). Also, the result shows the significant bias caused when animals travel faster than the observer [1] (pp. 131). To reduce bias in strip transect sampling, observers should record only those animals that were initially inside the strip and ignore those animals that enter from the side or behind [24]. As line transects are often much wider than strip transects and detection of animals is uncertain [25], it is not possible to exclude animals that enter the strip from the side, but often animals that overtake the observer can (and should) be excluded.

Bias in line transect sampling for most animal speeds is considerably less than that of strip transect sampling. The magnitude of the bias depends on the behaviour of the surveyed animal: those that quickly travel long distances cause more bias than those that travel slowly or remain within a small area [16]. Once again, survey procedure can reduce bias in line transect surveys by searching further to the side and a shorter distance ahead; however, this may increase the chances of failing to spot an animal before it moves in response to the observer [26] (pp. 129–131). If the animals to be surveyed tend to flee from the observer or be attracted, searching further along the line will reduce the bias caused by this responsive movement [1] (pp. 32). Clearly, this opposes our advice concerning independent animal movement. Often the bias caused by independent animal movement will be small compared to that caused by movement in response to the observer [27].

How to reduce bias from both independent and responsive movement will depend on the type of survey and species. For aircraft surveys, animal speed is typically slow relative to observer speed [28], and search effort is generally concentrated to the side rather than ahead. Thus independent animal movement is unlikely to cause bias. For shipboard surveys, observers tend to concentrate more effort on searching ahead rather than to the side. This has the advantage that animals are less likely to respond to the ship before detection, but greater bias will be generated by independent animal movement. Therefore ship speed should be such that bias from independent animal movement is low. For fast-moving animals, such as seabirds in flight, this may not be an option; then methods for correcting for movement should be considered. For example, seabirds on the sea might be surveyed, then a correction adopted based on the proportion of time birds are in flight. If seabirds don’t respond to the ship, then one strategy to eliminate bias is to simply record distances of detected birds when they pass abeam; if they do not pass abeam while visible, then they are not recorded [1] (pp. 198–203). Corrections have also been developed for seabirds in flight [15]. For terrestrial surveys of birds, surveying perched birds, and separately estimating the proportion of time birds are in flight, can be effective [29]. For surveys on foot of ground or arboreal animals, an assessment should be made of whether it is feasible for observers to travel sufficiently fast without compromising other assumptions, to ensure bias from independent animal movement is low. If not, corrections for animal movement should be considered.

Once the survey is completed there is, at present, no clear way to reduce bias in abundance estimates using the standard methods. For example, truncating the data [1] (pp. 103–108) has no discernible effect on the bias.

Here we have shown that bias can reach up to 10% for animal speeds of 50% – 80% of observer speed. This is useful for providing an idea of the severity of violating the assumptions of distance sampling. However, the quantities of bias presented here are dependent on the type of movement and survey protocol assumed. The expression for bias in strip transect sampling assumes a linear movement of animals, which is unrealistic and causes greater bias compared to other movement types [10]. Similarly, within the line transect simulation study we have not considered how variable animal speed or heterogeneous home range radius may affect bias. Furthermore, we only considered how the shape of the detection function affected bias by varying the shape parameter, which not only determines the function’s shape, but also the average probability of detection; it may be more informative to describe the relationship between detection function shape and bias for a fixed probability of detection. Also, in this study only the hazard-rate detection function was fit to the simulated data, while in practice other detection functions may be chosen by model selection [1] (pp. 110), which may affect bias.

In conclusion, independent animal movement can cause substantial bias in abundance estimates and its possible effects should be considered in line transect surveys. It arises from both the detection process inherent in line transect sampling and from the movement characteristics of the surveyed animal. There are, at present, no analytical methods to deal with this reality; however, bias is reduced by searching further perpendicularly to the line, searching less ahead and ignoring animals that overtake the observer.

## Supporting Information

S1 Code Computer Code for Simulation Study. (ZIP)
